# Nurse-patient interaction and self-transcendence: assets for a meaningful life in nursing home residents?

**DOI:** 10.1186/s12877-020-01555-2

**Published:** 2020-05-07

**Authors:** Gørill Haugan, Britt Moene Kuven, Wenche Mjanger Eide, Siv Eriksen Taasen, Eva Rinnan, Vivien Xi Wu, Jorunn Drageset, Beate André

**Affiliations:** 1 Department of Public Health and Nursing, Faculty of Medicine and Health Science, NTNU Norwegian University of Science and Technology, Trondheim, Norway; 2 NORD University, Faculty of Nursing and Health Science, Levanger, Norway; 4 Trondheim Municiaplity, Trondheim, Norway; 3 Faculty of Health and Social Science, Western University of Applied Science, Bergen, Norway; 5 Alice Lee Centre for Nursing Studies, Yong Loo Lin School of Medicine, The National University of Singapore, Singapore, Singapore; 6 Department of Global Health and Primary Care, University of Bergen, Bergen, Norway

**Keywords:** Interpersonal and intrapersonal self-transcendence, Meaning-in-life, Nursing, Nurse-patient interaction, Nursing home, Older adults, Wellbeing

## Abstract

**Background:**

Due to the shift to an older population worldwide and an increased need for 24-h care, finding new and alternative approaches to increase wellbeing among nursing home (NH) residents is highly warranted. To guide clinical practice in boosting wellbeing among NH residents, knowledge about nurse-patient interaction (NPI), inter- (ST1) and intra-personal (ST2) self-transcendence and meaning-in-life (PIL) seems vital. This study tests six hypotheses of the relationships between NPI, ST1, ST2 and PIL among cognitively intact NH residents.

**Methods:**

In a cross-sectional design, 188 (92% response rate) out of 204 long-term NH residents representing 27 NHs responded to NPI, ST, and the PIL scales. Inclusion criteria were: (1) municipality authority’s decision of long-term NH care; (2) residential time 3 months or longer; (3) informed consent competency recognized by responsible doctor and nurse; and (4) capable of being interviewed. The hypothesized relations between the latent constructs were tested through structural equation modeling (SEM) using Stata 15.1.

**Results:**

The SEM-model yielded a good fit (χ2 = 146.824, *p* = 0.021, df = 114, χ2/df = 1.29 RMSEA = 0.040, p-close 0.811, CFI = 0.97, TLI = 0.96, and SRMR = 0.063), supporting five of the six hypothesized relationships between the constructs of NPI, ST1, ST2 and PIL.

**Conclusion:**

NPI significantly relates to both ST1, ST2 and PIL in NH residents. ST revealed a fundamental influence on perceived PIL, while NPI demonstrated a significant indirect influence on PIL, mediated by ST.

## Background

Currently, the world faces a rapidly aging population. The document An Aging World [1] highlights a shift to an older population worldwide and its consequences: the population aged 80+ grows faster than any younger age group, and those aged 100+ are growing fastest [2]. This shift started in high-income countries. However, today most people in the world can expect to live into their sixties and beyond [3]. During the period from 2015 and 2050, the proportion of those 60 years and more will nearly double from 12 to 22%; by 2050, individuals aged 60 years and older is expected to total 2 billion, up from 900 million in 2015 [1, 3]. Currently, low- and middle-income countries are experiencing the greatest demographic change; hence, all countries are now facing major challenges to ensure that their health and social systems are ready to make the most of this demographic shift [3].

As longevity is increasing worldwide, it is important to ensure that the extra years of life are worth living, despite chronic illnesses. However, still evidence do not suggest that older people today are experiencing their later years in better health than their parents [4, 5]. Increased age is followed by an increased incidence of functional and chronic comorbidities and diverse disabilities [6]. Hence, a noteworthy part of older adults will need 24-h nursing home (NH) care. In Norway, about 7.2% of the people older than 67 years and 9.1% of those older than 80 years live their last phase of life in NHs; mean residential time is about 1–2 years [7, 8], and about 30–40% of deaths annually in the US and Norway happen in NHs [8, 9]. Hence, the NH population is characterized by high age, frailty, chronic illnesses, multiple diagnoses, mortality, disability, powerlessness, dependency, vulnerability, poor general health and a high symptom burden [10–12]. Health-promotive initiatives enhancing wellbeing among older persons living in NHs will become ever more important in the years to come.

Accordingly, relocating to a NH is caused by numerous losses, illnesses, disabilities, loss of functions and social relations, and facing the end-of-life, all of which increases an individual’s vulnerability and distress. Loneliness and depression are identified as risks to older people’s emotional wellbeing [13–15]. Consequently, this population is at a high risk of declined meaning-in-life and wellbeing [16, 17]; thus, finding new and alternative approaches to increase wellbeing among older adults in NHs is highly warranted.

Spirituality has been perceived as a major resource for wellbeing in late life [18], particularly among frail and vulnerable older people such as NH residents [19]. As an essential aspect of spirituality, the concept of meaning-in-life is commonly addressed in the nursing literature [20] and is seen to be of importance to wellbeing in older adults [17, 18, 21, 22], in NHs [23–25], and at the end of life [26–28]. Research implies that perceived meaning-in-life is important for maintaining not only mental/emotional wellbeing, but physical and functional wellbeing as well [11, 29]. A novel study demonstrates humans’ holistic existence showing that life meaning as well as loneliness affected older adults’ brain function [30]. These findings advance our understanding of phenomena such as meaning-in-life and loneliness which operate not only by emotions or experiences but represent physical states in the human brain (ibid.).

Social relationships imbue life with meaning, whereas loneliness diminishes one’s sense of meaning-in-life. The ‘search for meaning and finding answers’, ‘feelings of support and trust’, ‘a perspective beyond death’ [19], along with a sense of belonging [31–34] are fundamental to perceived meaning-in-life, spiritual-emotional wellbeing [35, 36] and life satisfaction [37] in older adults. Perceived meaning-in-life is seen to predict life satisfaction among NH residents [38].

Additional to meaning-in-life, the concept of self-transcendence addresses an enhanced understanding of wellbeing in late adulthood [39, 40] and among different vulnerable populations, such as NH residents, cancer and AIDS patients as well as homeless individuals (ibid.). The human developmental theory emphasises maturity as the developmental task across the life-span [41]; self-transcendence is defined as a “characteristic of developmental maturity wherein there is enhanced awareness of the environment and an orientation toward broader life perspectives” [39]. Self-transcendence is salutogenic; it is conceptualized as an inherent resource for wellbeing, particularly in challenging health conditions including end-of-life. Nursing’s role is to describe, explain, and facilitate these processes as they occur in human beings during health experiences and events across the lifespan [40]. Further, self-transcendence is seen to be a powerful coping mechanism involving adaption to physical, emotional and spiritual distress, leading to personal transformation, maturity and wellbeing. Self-transcendence is positively related to mental/emotional wellbeing, health and functioning in adults confronting personal mortality because of advanced age and/or enduring illness [42–46]. Connectedness is the core of self-transcendence [39, 40].

The experience of connectedness for older people in long-term care settings is linked with quality-of-life (QoL) and successful aging [47]. Research has shown that self-awareness, meaningful relationships with family and friends, involvement in meaningful activities and connections with wider society are fundamental prerequisites of connectedness for older people [47]. However, barriers to these prerequisites are evident for many residents in long-term care settings [47]. Largely, the nurse-patient relationship represents the main resource for connectedness while staying in an NH. Therefore, the nurse-patient interaction might be crucial for wellbeing in NHs. Self-transcendence and meaning-in-life have demonstrated significant relations with both physical, emotional, social, functional [29, 48] and spiritual [49] wellbeing among cognitively intact NH residents, indicating that enhancing self-transcendence and meaning-in-life positively influences on all aspects of wellbeing.

To summarise, the literature suggests that nurse-patient interaction, self-transcendence, and meaning-in-life are vital to wellbeing among older adults in NHs. Thus, we expected meaning-in-life and self-transcendence to be correlated, and that nurse-patient interaction would influence on both constructs. To get further insights into how self-transcendence and meaning-in-life relate with each other, as well as with nurse-patient interaction, this study investigates the associations between nurse-patient interaction, self-transcendence and perceived meaning-in-life. Such knowledge can guide clinical practice in how to best and efficiently boost wellbeing among older adults in NHs.

## Aims and hypotheses

The present study was therefore designed to investigate the relationships between nurse-patient-interaction, self-transcendence and meaning-in-life among cognitively intact NH residents utilizing structural equation modeling (SEM). The research questions were: (1) Does the nurse-patient interaction affect interpersonal self-transcendence (ST1), intrapersonal self-transcendence (ST2) and meaning-in-life in cognitively intact NH residents? and (2) How do the constructs of ST1, ST2 and meaning interrelate?

Psychometric studies have revealed a two-factor construct (ST1, ST2) showing the best fit for self-transcendence [50], whereas nurse-patient interaction [51] is found to be a one-dimensional construct. Thus, the two-factor construct of self-transcendence (where ST1 embraces interpersonal aspects whereas ST2 covers intrapersonal aspects) along with the one-factor models of nurse-patient interaction [51] and meaning-in-life (PIL) [52] were applied in the present study. Based on the theoretical and empirical knowledge of nurse-patient interaction, self-transcendence (ST) and meaning-in-life, the following hypotheses were formulated:

- Hypothesis 1 (H1): Nurse-patient interaction positively affects ST1 (Inter-personal ST).
- Hypothesis 2 (H2): Nurse-patient interaction positively affects ST2 (Intra-personal ST).
- Hypothesis 3 (H3): Nurse-patient interaction positively and directly affects meaning-in-life (PIL).
- Hypothesis 4 (H4): Nurse-patient interaction indirectly affects meaning-in-life (PIL).
- Hypothesis 5 (H5): ST1 positively affects PIL.
- Hypothesis 6 (H6): ST2 positively affects PIL.

**Fig. 1:**
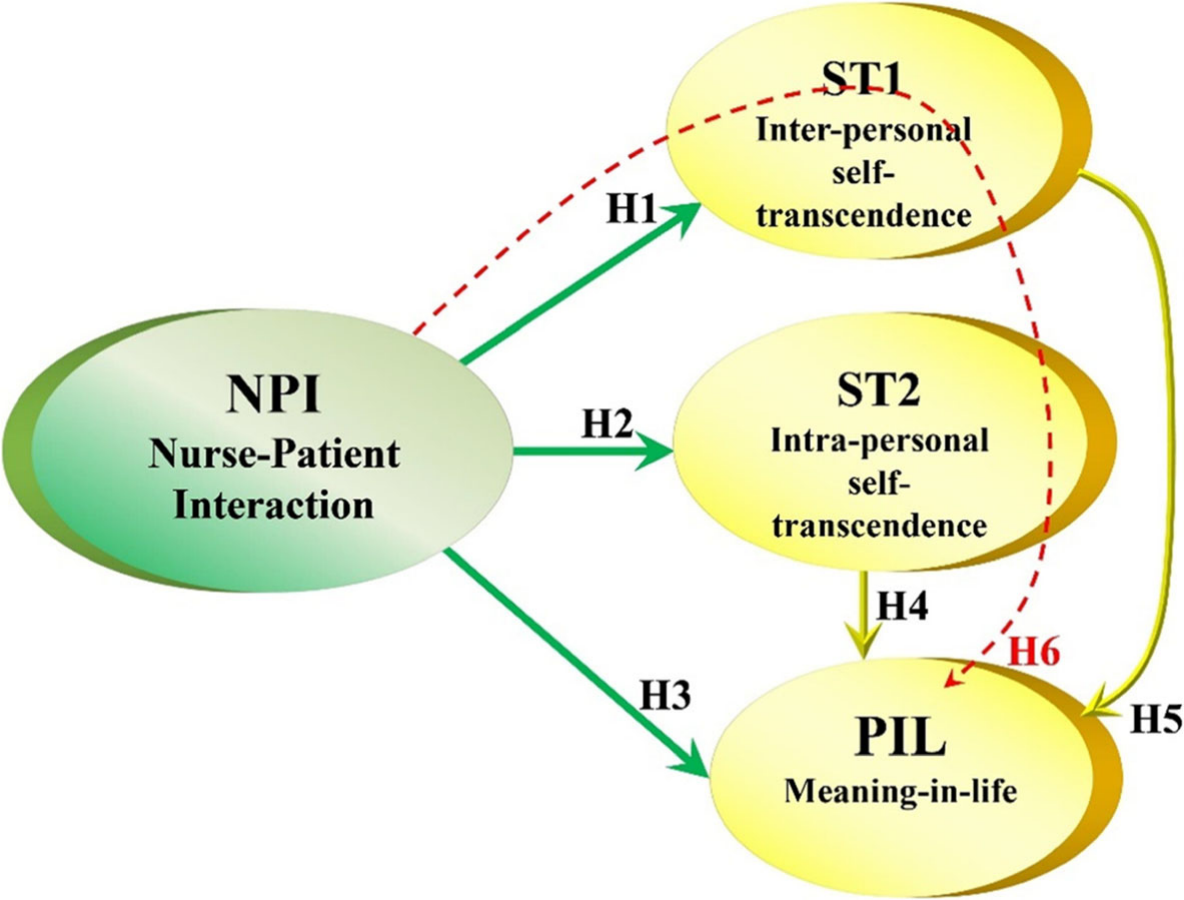
Hypotheses tested

A hypothesized structural equation model (SEM) with bases in existing theory [39, 40, 53, 54] and previous empirical research [29, 31, 48, 55] was tested. Figure 1 shows the hypotheses representing the relationships implying the influences between the latent constructs in the model. Significant associations have been demonstrated between nurse-patient-interaction and self-transcendence [51] and meaning [36]. H1-H4 in Fig. 1 reflects the hypothesized associations between nurse-patient interaction, self-transcendence and meaning. ST1 and ST2 involve aspects such as having interests and hobbies, involving and caring for others (ST1), adapting well and self-acceptance (ST2); hence, these dimensions were hypothesized to associate with meaning-in-life (PIL), shown as hypotheses H5 and H6 in Fig. 1.

By doing so, we pursued contributing to a nursing perspective of promoting the wellbeing of older adults in NHs in three ways. First, research focusing on the dimensions of self-transcendence, meaning and nurse-patient interaction in NHs is scarce [56]. The health-promoting magnitude of these dimensions to older adults and NH patients has recently been identified [31, 38, 57–59], the fundamental relationships between nurse-patient-interaction, self-transcendence and meaning in NH residents are scarcely documented, though. Second, examining the associations between self-transcendence and meaning contributes to a deepened theoretical understanding of how nursing care can boost NH residents’ wellbeing. Accordingly, this study provides more specific insights about the mechanisms involved in the relationships between these vital dimensions, as well as on nurse-patient interaction’s associations to them. As self-transcendence and meaning are found to be interwoven with individuals’ health and wellbeing [60–62], nurse-patient interaction might influence not only on NH residents’ emotional and social wellbeing, but also on their physical wellbeing [48, 63–67].

## Methods

### Participants

For running SEM-analyses, large samples of *N* ≥ 200 are recommended [68–71]. Thus, the present sample consisted of 188 (92.0%) out of 204 long-term NH residents, residing in 27 NHs, located in two small and one large urban municipality in Mid-Norway (*N* = 88, living in Joy-of-Life Nursing Homes (JoLNH); these are NHs which have undergone a certification process of 1–2 year working on meeting the residents social, cultural and spiritual needs [72], along with a large urban municipality in Western Norway (*N* = 100, ordinary NHs which are not certified as a JoLNH). Long-term NH care was termed as 24-h care. Exclusion criteria were short-term care, rehabilitation stay, and diagnosed with dementia. Inclusion criteria were as follows: (1) municipality authority’s decision of long-term NH care, (2) residential stay of 3 months or more (the reason for this was two-fold; 1) moving to an NH represents stress and is found to be demanding; therefore, we did not want to interrupt asking many questions about wellbeing, joy-of-life, meaning-in-life, etc. during this vulnerable phase, and 2) before replying to the questionnaire the residents need some experience of the inhouse life in an NH. Thus, we wanted the resident to have stayed for 3 months or more), (3) informed consent competency recognized by responsible doctor and nurse, and (4) capable of taking part adequately in an interview situation.

### Procedures

Cross-sectional data were collected during 2017 and 2018. The Regional Committee for Medical and Health Research Ethics in Norway (ref.nr 2014/2000/REK Central) as well as the Management Units at the 27 NHs approved the study. The participants provided voluntarily written informed consent. A nurse who the NH residents knew well presented them with oral and written information about their rights as participants and their rights to withdraw at any time. Each participant provided informed consent. This population may have difficulties completing a questionnaire on their own. Therefore, six trained researchers (three in each part of Norway) conducted one-on-one interviews in private. Researchers with an identical professional background (RN, MSc, trained and experienced in communication with the elderly, as well as teaching gerontology at an advanced level) were trained to conduct the interviews in the same manner. To avoid misunderstandings, interviewers read each question loudly, and held a large-print copy of questions and possible responses in front of the participants. This study is part of the project Health promotion factors in Joy-of-Life Nursing Homes funded by the Norwegian Research Council. This project aimed to explore health promotion factors in Norwegian NHs, including certified JoLNHs and ordinary NHs. Accordingly, the three scales used in this study were part of the larger questionnaire comprising 9 scales representing 120 items as well as sociodemographic data (age, gender, marital status, residential time in the NH); thus, small breaks at specific points during the interview process were adopted to avoid tiring the participants. The nine scales were assessed in the following order; 1. Health-related QoL assessing symptom burden, 2. Joy-of-life, 3. a single item assessing loneliness, 4. Sense of coherence, 5. Self-transcendence, 6. Meaning-in-life, 7. Nurse-patient interaction, 8. Depression and anxiety, and 9. OPQOL-brief quality-of-life questionnaire. The OPQOL-brief data were recently published in a study testing the psychometric properties of the Norwegian version of OPQOL-brief [73]. Similarly, the Joy-of-Life scale was psychometrically tested, and used in a study assessing the associations between Nurse-Patient Interaction and joy-of-life [74] in the sample which is used for the present study.

### Measures

The Nurse-Patient-Interaction Scale (NPIS) was developed in Norway to assess the vital characteristics of NH residents’ experiences of the nurse-patient interaction [51]. The NPIS 14 items assess NH resident’s ability to obtain a sense of wellbeing from essential relational and caring qualities embedded in the nurse-patient interaction. The term ‘nurse-patient interaction’ covers NH residents’ perceived interaction with their professional caregivers in the NH; these professionals are nurses (RNs), Licensed Nurse Assistants (LNAs) and caregivers without any formal education in health care. The NPIS is a 10-point scale from 1 (not at all) to 10 (very much); higher numbers indicate that residents perceive better nurse-patient-interaction. Examples of NPIS-items include the experience of being taken seriously, and being understood, respected, and recognized as a person, as well as being listened to and feeling good resulting from the nurse-patient-interaction. The items were developed to measure NH residents’ ability to derive a sense of wellbeing through the nurse-patient-interaction. The NPIS has shown good psychometric properties with good construct validity and reliability among NH residents [51].

Self-transcendence was assessed by the Self-Transcendence Scale (STS), developed in the U.S. to identify experiences of later life and reflecting expanded boundaries of the self [39, 75]. The STS comprises 15 items rated on a 4-point Likert-type scale from 1 (not at all) to 4 (very much); higher scores indicate higher self-transcendence. The STS was translated into Norwegian and validated among NH residents, showing a two-factor construct of self-transcendence [50], which is used for the present study. The items ‘Having hobbies or interests I can enjoy’, ‘Involving in others’ and ‘Sharing my wisdom’, were indicators for interpersonal self-transcendence (ST1). Test items for intrapersonal self-transcendence (ST2) were ‘Accepting myself as I grow older’, ‘Adjusting well to my present life situation’ and ‘Adjusting to changes in my physical abilities’. In former studies, Cronbach’s α for the total scale ranges between 0.80–0.88 [76–78].

The Purpose-in-Life-Test (PIL) was designed to assess meaning-in-life and is commonly used for this purpose [79–81]. The PIL contains 20 items which are based on Frankl’s logotherapy [82–84]. Test items include questions such as ‘My life is: empty, filled only with despair – running over with exciting things’ and ‘In thinking of my life, I: often wonder why I exist – always see reasons for being here.’ Each of the 20 statements is scored from 1 to 7 where 4 represents a neutral value; whereas the numbers from 1 to 7 stretch along a continuum from one extreme feeling to the opposite kind of feeling. The range of possible scores is 20–140; numerically higher scores indicate greater meaningfulness [82]. The PIL was translated into Norwegian by Bondevik [85] and has previously been used and validated with older adults showing good psychometric properties [52, 86–88].

### Statistical analysis

The data were analyzed by descriptive statistics using IBM SPSS version 25. The hypothesized relations between the latent constructs of NPIS, ST1, ST2 and PIL were tested through a structural equation model (SEM) using Stata 15.1 [89]. Using SEM accounts for random measurement error and the psychometric properties of the scales involved are more accurately derived. Missing data was low in frequency and were handled employing the listwise procedure. Research has indicated that Cronbach’s α cannot be generally relied on as an estimator of reliability [90]. Thus, composite reliability was estimated using the formula by Hair and colleagues [70], as shown in Table 2; a coefficient of ≥0.7 is good whereas 0.6 is considered fair for both reliability coefficients [77, 79, 82, 83]. For the correlation analyses, the *p*-value was set to 1%, whereas the estimates based in SEM-analyses commonly include both 5 and 1% *p*-values. Factor loadings below 0.32 are considered poor, ≥0.45 fair, ≥0.55 good, ≥0.63 very good, and above 0.71 are excellent [91].

#### Model fit

In line with the rule of thumb of conventional cut-off criteria [92] the following fit indices were used to evaluate model fit: chi-square (χ^2^) and its p-value which is significant in most cases. Therefore, instead of solely considering the *p*-value, it is suggested to consider the value of χ^2^/degrees of freedom (df), which should be ≤2 for good fit and ≤ 3 for an acceptable fit [93]. Since skewness and kurtosis were significant, the Satorra-Bentler-scaled chi-square statistic is the correct asymptotic mean and was therefore applied as a goodness-of-fit statistic [94]. However, the χ^2^statistic is sensitive to sample size and is therefore not relied upon as a basis for acceptance or rejection of the model [92, 95]. As a result, the use of multiple fit indexes has developed to provide a more holistic view of the goodness of fit, taking account not only of sample size but also of model complexity and other relevant issues of the study. Further, the root mean square error of approximation (RMSEA) and the standardized root mean square residual (SRMS) with values below 0.05 indicating good fit, whereas values smaller than 0.08 are interpreted as acceptable [92, 96]. Also, the comparative fit index (CFI) and the Tucker Lewis Index (TLI) were used with an acceptable fit at 0.95/0.90 respectively, and good fit at 0.95/0.97 and above (ibid.).

Before examining the hypothesized relationships, the measurement models were tested by confirmatory factor analysis (CFA) using Stata 15.1 [89]. An appropriate power analysis is dependent on the ratio between the total number of variables (error measurements, observed and latent variables) and the sample size; one observed variable per 10 participants is given as a rule of thumb [68, 69, 71]. Thus, to reduce model complexity, the measurement model for nurse-patient-interaction was tested by CFA; by considering the loadings, R^2^-values (items which explain very little of the factor is dismissed) as well as covering the breadth and nuances of the actual construct, the indicator variables were reduced to six for NPIS (χ^2^ = 8.850, *p* = 0.451, df = 9, RMSEA = 0.000, p-close 0.748, CFI = 1.00, TLI = 1.00, SRMR = 0.021), while meaning-in-life (χ^2^ = 5.066, *p* = 0.408, df = 5, RMSEA = 0.008, p-close 0.643, CFI = 0.999, TLI = 0.999, SRMR = 0.030), were represented by five indicators. Furthermore, for the ST-construct (χ^2^ = 3.886, *p* = 0.867, df = 8, RMSEA = 0.000, p-close 0.961, CFI = 1.00, TLI = 1.00, SRMR = 0.022) the three-indicator rule [70] was applied, including three indicators for ST1 and ST2, respectively, (totally six indicators). Consequently, the total number of indicators included in the SEM-model was 17 (6 + 5 + 3 + 3).

## Results

### Descriptive analysis

The 188 participants’ ages ranged between 63 and 104 years, with a mean age of 87.4 years (SD = 8.57). With 8 missing, the effective sample was *N* = 180, consisting of 132 women (73.3%) and 48 men (26.7%). The mean age for women was 88.3 years (SD = 1.80) and 86 years (SD = 1.16) for the men. In total, 23 were married, 22 cohabitating, 1 was single, 106 were widows/widowers, and 36 were divorced. Table 1 displays the means (M), standard deviations (SD), Cronbach’s α, and Pearson’s correlation matrix for the latent study variables. The correlations between the measures were in the expected direction. Moderate correlations were found between the latent constructs (Table 1). The α-levels for the various measures indicate an acceptable level of inter-item consistency with Cronbach’s alpha coefficients ranging between 0.63–0.88 [97]; however, ST1 and ST2 comprised only three items each and consequently displayed a lower α coefficient of .63 (Table 1).

**Table 1:** Mean, Cronbach’s alpha, and correlation coefficients for the study variables

Construct	Mean (sd)	Items	Cronbach’s Alpha	^a^ST1	^b^ST2	^c^PIL	^d^NPIS
ST1	2.54 (.711)	3	0.63	1			
ST2	3.20 (.675)	3	0.78	.26**	1		
PIL	4.11 (.613)	5	0.71	.37**	.27**	1	
NPIS	7.98 (1.91)	6	0.88	.30**	.33**	.19**	1

** *p*-value < 0.01, ^a^*ST1* Interpersonal self-transcendence, ^b^*ST2* Intrapersonal self-transcendence, ^c^*PIL* Meaning-in-life, ^d^*NPIS* Nurse-Patient Interaction. Listwise *N* = 180, Missing *N* = 8

### Model testing and model fit

#### SEM-analyses

To investigate how the nurse-patient-interaction related to ST1, ST2 and meaning, as well as how the dependent latent variables were inter-related, a SEM-model comprising 17 indicators was estimated. For scaling the variances of the dependent latent variables were set at 1. Table 2 lists the measurement models with factor loadings, t-values, R^2^-values, and composite reliability. All factor loadings were significant, showing fair to good estimates ranging from 0.48 to 0.87, with R^2^-values between 0.23 and 0.76. Composite reliability ranged between 0.63–0.80, with ST1 and PIL showing the lowest coefficients of 0.63 and 0.64, respectively (Table 2).

**Table 2:** Measurement models for Nurse-Patient Interaction (NPIS), Self-transcendence (ST1 and ST2) and Meaning-in-Life (PIL)

Items	Parameter	Stata Estimate^**b**^	t-value	Bentler-Raykov squared multiple correlation^c^ R^**2**^
**NPIS Nurse-Patient Interaction**				
^e^NPIS3	λx3,1	0.80	24.80^a^	0.64
NPIS4	λx4,1	0.73	18.16^a^	0.53
NPIS5	λx5,1	0.77	21.23^a^	0.58
NPIS11	λx11,1	0.79	23.79^a^	0.63
NPIS13	λx13,1	0.84	28.89^a^	0.70
NPIS14	λx14,1	0.52	08.90^a^	0.27
**ST1 Interpersonal Self-Transcendence**				
^f^ST1	*λx1,2*	0.56	7.64^a^	0.31
ST3	*λx3,2*	0.56	7.94^a^	0.31
ST6	*λx6,2*	0.72	10.81^a^	0.52
**ST2 Intrapersonal Self-Transcendence**				
ST2	*λx2,3*	0.54	8.89^a^	0.29
ST4	*λx4,3*	0.87	20.32^a^	0.76
ST5	*λx5,3*	0.82	18.49^a^	0.67
**PIL Meaning-in-life**				
^g^PIL5	*λy11,2*	0.48	6.46^a^	0.23
PIL8	*λy11,2*	0.49	6.51^a^	0.24
PIL10	*λy12,2*	0.53	7.23^a^	0.29
PIL18	*λy11,2*	0.54	7.76^a^	0.30
PIL20	*λy13,2*	0.54	7.60^a^	0.29
^d^*ρ*_*c*_ST1	^d^*ρ*_*c*_	0.63		
*ρ*_*c*_ST2	*ρ*_*c*_	0.80		
*ρ*_*c*_PIL	*ρ*_*c*_	0.64		
*ρ*_*c*_NPIS	*ρ*_*c*_	0.89		

^a^Significant at the 1% level. ^b^Completely Standardized Factor Loadings. ^c^The Bentler-Raykov squared multiple correlation coefficient = R^2^. ^d^Composite reliability $\boldsymbol{\rho} c=\frac{{\left(\sum \uplambda \right)}^2}{{\left(\sum \uplambda \right)}^2+\sum \left(\uptheta \right)}$

^e^*NPIS* Nurse-Patient Interaction Scale item. ^f^*ST* Self-Transcendence scale item, ^g^*PIL* Purpose-In-Life Test item. Listwise, *N* = 180, Missing *N* = 8, 17 items included

**Fig. 2:**
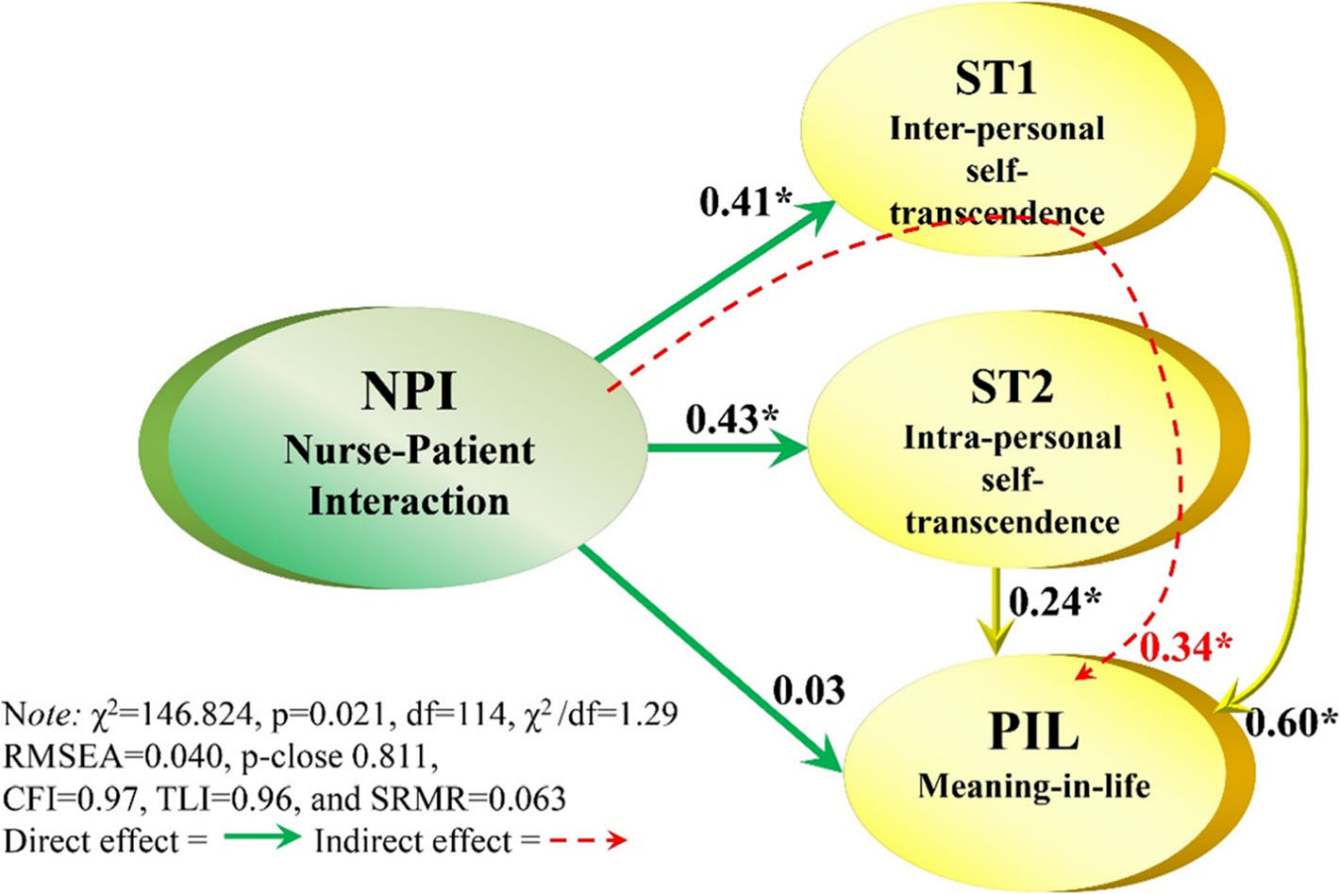
The SEM-model testing the hypotheses H1-H6. Direct and indirect associations. *significant at the 5% level

Figure 2 portrays the SEM-model showing the structural regression coefficients and the fit indices.

The SEM-model yielded a good fit to the data (χ^2^ = 146.824, *p* = 0.021, df = 114, χ^2^ /df = 1.29 RMSEA = 0.040, p-close 0.811, CFI = 0.97, TLI = 0.96, and SRMR = 0.063). Table 3 shows the standardized regression coefficients of the directional relationships, as well as the total and indirect effects between the latent constructs in the SEM-model. According to Fig. 2, the hypotheses H1 and H2 were significant showing directional paths from nurse-patient-interaction to ST1 (γ1,1 = 0.41) and ST2 (γ2,1 = 0.43), while the path from NPIS on PIL (H3) was not significant. Furthermore, the direct relationships (Table 3) between the dependent variables, as assumed in H5 and H6 displayed significant values from ST1 to PIL (β1,3 = 0.56) and from ST2 to PIL (β2,3 = 0.30). Looking at the indirect influences (Table 3, Fig. 2), nurse-patient-interaction revealed a significant indirect effect on PIL (H4), mediated by ST1 and ST2 (0.34, total effect 0.37) (Table 3). Thus, the hypotheses H1, H2, H4-H6 were supported.

**Table 3:** SEM-model. Standardized Stata estimates of direct and indirect relationships and total effects of Nurse-Patient-Interaction (NPIS), on Self-Transcendence (ST1 and ST2) and Meaning-in-life (PIL)

Construct	Parameter		
*Direct relationships between NPIS and the dependent latent constructs*			
		**NPIS**^**c**^	
ST1^d^	γ^b^ 1,1	0.41	
	t-value	3.59^a^	
ST2^e^	γ 1,2	0.43	
	t-value	4.37^a^	
PIL^f^	γ 1,3	0.031	
	t-value	0.27	
*Indirect*^h^*(mediated) relationship between NPIS and PIL*			
		**NPIS**	**TOTAL EFFECT**^**i**^
PIL	γ 1,3	0.34	0.37
	t-value	3.13^a^	3.13^a^
*Direct relationships between the dependent latent constructs*			
		**PIL**	
ST1	β^g^ 1,2	0.60	
	t-value	3.47^a^	
ST2	β 3,5	0.24	
	t-value	2.10^a^	

^a^Significant at the 5% level. Listwise *N* = 180, Missing *N* = 8. ^b^Gamma (γ); standardized regression coefficients representing directional relationships between ^c^*NPIS* Nurse-Patient-Interaction and ^d^*ST1* Interpersonal Self-Transcendence, ^e^*ST2* Intrapersonal self-transcendence and ^f^*PIL* Meaning-in-life). ^g^*Beta (β)* standardized regression coefficients representing directional relationships between ST1, ST2 and PIL. ^h^Indirect effects represent the influence of NPIS mediated by intervening variables (mediators). ^i^Total Effect represent the total influence of the explanatory variable NPIS (direct + indirect effects)

## Discussion

The present study aimed to explore 1) the influences of NPIS on ST1, ST2, and PIL, and 2) the associations between ST1 and ST2 on PIL in cognitively intact NH residents. Among the six hypotheses tested five were supported at the 5%-level. We could show significant associations between NPIS on ST1, ST2 as well as a mediated association on PIL. Additionally, our results showed a statistically significant association between ST1 and ST2 on PIL.

### Nurse-patient-interaction relates to self-transcendence and meaning-in-life

More specifically, a significant impact of nurse-patient-interaction on interpersonal (ST1), intrapersonal self-transcendence (ST2) and meaning-in-life was found, as well as a significant indirect relation to meaning-in-life, mediated by self-transcendence (ST1, ST2).

Our results showed that NPIS seems to have an outstanding position as a resource for ST1, ST2 and meaning-in-life in this vulnerable population. However, the influence on PIL was mediated by ST1 and ST2, indicating that to facilitate NH residents’ meaning-in-life, health professionals should support ST1 and ST2. Previous research underlines that NH residents’ perceived nurse-patient interaction is critical to their sense of dignity, self-respect, feelings of self-worth, meaning-in-life, and wellbeing [33, 36, 59, 98–100]. NPIS covers aspects such as being taken seriously, being understood, respected and recognised as a unique person; all of which relate closely to a sense of dignity, as well as a sense of self-worth and self-respect. Resulting from their frailty, vulnerability and dependency, NH residents stress their need for connectedness or belongingness with the nurses [31–33, 101, 102] highlighting the relationships to their caregivers as essential for wellbeing [103, 104]. Moreover, dignity significantly predicts older adults’ satisfaction with NH staff [38] and has been related to the nurse-patient relationship [33]. NPIS emerged as vital for self-transcendence in the present results as well as in the literature [57, 105–108], and is found to boost global wellbeing [29, 48]. Consequently, nurse-patient interaction might influence on NH residents’ wellbeing; physically, emotionally, socially, functionally and spiritually.

Former studies have shown that NH residents emphasize the nurses’ attitudes, appearance and behaviours [108, 109], to act as a confirmation of their worthiness or worthlessness [31, 110, 111]. Connectedness in a trusting nurse-patient relationship reduce anxiety and depressive symptoms [112], and facilitate feelings of being valuable, safe and cared for. Such experiences provide meaning-in-life while facing the end of it. Along with competent pain and symptom management, the nature of the nurse-patient interaction in long-term NH care is crucial. Frustration, suffering, hopelessness, meaninglessness and depression result from not being attended to or treated with indifference, and thereby violating individuals’ sense of worthiness [113, 114] which negatively impact residents’ mental and physical symptoms or ailments [29, 112].

Possibly, older adults’ self-acceptance and thereby their sense of self-worth and value can be strengthened by active listening, recognizing and empowering the uniqueness of this person whose subjective experiences are taken seriously and respected, all of which supporting global wellbeing. If older adults’ in NHs feel understood, acknowledged, confirmed, and valued by their nurses, self-transcendence, and meaning will increase; consequently, gratefulness and wellbeing redoubles. However, the accomplishment of such a health-promoting nurse-patient interaction requires caregivers who are willing to and competent in engaging with their residents in different ways, such as learning about the person through life histories [115–118], listening to their life experiences, wisdom, dreams and frightens. Professional nursing care is determined by the way nurses use their knowledge, attitudes, behaviour, and communication skills to appreciate the uniqueness of the person being cared for [118–120].

### The interrelations between self-transcendence and meaning

The second research question aimed at investigating the inter-relationships between the constructs of inter- and intra-personal self-transcendence and meaning-in-life. The latter is often seen to be an explicit goal of NH care in Norway [29, 31, 121]. If looking at the total and indirect effects, interpersonal self-transcendence (ST1) showed a great influence on meaning-in-life; ST1 includes being involved with others and the society, having hobbies or interests and sharing one’s wisdom with others [50]. This finding is in line with a longitudinal study by Norberg and her colleagues (2015) of 190 oldest-old individuals in Sweden; self-transcendence was significantly related to wellbeing overall. Moreover, studies among NH residents in Taiwan and Norway have disclosed a negative association with depressive symptoms [122, 123] and a positive association with wellbeing [48]. Also, Hoshi [124] found a mediated effect of self-transcendence on the relationship between vulnerability and wellbeing in 105 Japanese hospitalized elders. Accordingly, facilitating these aspects among NH residents will increase meaning-in-life, and consequently wellbeing. Furthermore, intrapersonal self-transcendence (ST2) involving self-acceptance, adapting well to this specific life situation and one’s functionality, displayed a significant impact on meaning. Research reveals that self-transcendence reduces stress, enhances wellbeing, hope and meaning among several patient groups facing the vulnerability of serious, progressive disease including multiple sclerosis and systemic lupus erythematosus [125], older women living with rheumatoid arthritis [126] as well as among individuals with spinal muscular atrophy [127] and amyotrophic lateral sclerosis [128]. Also, self-transcendence has been used to design programs effective in promoting successful aging among older adults in the community [129, 130]. This indicates that supporting self-acceptance and adaption to one’s life situation will support meaning among older adults in NHs. The various aspects of self-transcendence seemed to be forceful vitalities, revealing significant influences on meaning-in-life, which represents an essential goal for NH care.

## Strengths and limitations

A notable strength of this research is the empirical examination of associations of various constructs that are scarcely elucidated. This study expands previous research by testing the associations between nurse-patient interaction, self-transcendence and meaning-in-life in an NH population utilizing structural equation modeling. The SEM measurement technique includes estimates for random measurement error, thus the involved measurement models (here models for NPI, ST1, ST2 and PIL) are more precisely derived. The study builds on a strong theoretical foundation with the use of scales demonstrating good psychometrical properties. Nevertheless, the present findings must be discussed with some limitations in mind.

The SEM-model tested comprises 17 variables, indicating a desirable sample size of minimum *N* = 170 [69–71]. In the present study listwise N was 180, which should be enough. Still, a larger sample would significantly strengthen the statistical power of the tests. Information input to the SEM estimation increases both with more indicators per latent variable, and with more sample observations. More indicators per latent variable would have strengthened the composite reliability and the Cronbach’s α but weakened the statistical power. Therefore, with reference to sample size we reduced the indicators for each of the latent constructs. Nevertheless, composite reliability was acceptable to good, with ST1 (three items), showing the lowest reliability.

The cross-sectional design of this study implies that we cannot make conclusions on the causality. That is, we cannot define the direction of the paths with certainty [70]. Feasibly, the latent variable performs both as a predictor and an outcome of another construct. Despite a good fit, some alternative model might possibly fit better or be more accurate. However, the fit indices and composite reliability underpin the present results. There was also no problem with discriminant and convergent validity as we found good factor loadings indicating that the theoretical plausibility was good; all paths corresponded well to the theoretical basis, which supports the findings.

The fact that the researchers visited the participants to help fill in the questionnaires might have introduced some bias into the respondents’ reporting. The three scales used were part of a larger questionnaire comprising nine scales and 120 items. Thus, frail, older NH residents might tire when completing the questionnaires, representing a possible bias to their reporting. To avoid such a bias, experienced researchers were carefully selected and trained in conducting the interviews following a standardized procedure including taking small breaks at specific points during the process. Moreover, the order of the scales might affect the participants’ reporting; in this study demographics were collected firstly, followed by symptom burden, joy-of-life, loneliness (one item), sense of coherence, ST, PIL, NPI, anxiety and depression, and global QoL. This order was used in every interview, including a 5 minutes break between ST and PIL to avoid tiring the respondent. Since residents with dementia, as well as short time stay and rehabilitation residents were excluded in this study, the present results cannot be generalized to the entire NH population.

## Implications to nursing practice

The results of this study accentuate that a holistic and person-centered care based in a health-promoting nurse-patient interaction is recommended [115, 116, 131]. However, NH staff members in general are not well trained in nurse-patient-interaction. There is a need of educational nursing curricula emphasizing knowledge about and training in nurse-patient interaction as a resource for wellbeing, mediated by self-transcendence and meaning. Also, NH caregivers should be provided further education and support [132–134]. Appropriate learning programs facilitating health professionals’ interacting skills should be employed along with assessing their usefulness [135, 136]. Additionally, NH staff members have limited time along with an experience of minor autonomy in their job performance. Research discloses that health professionals in NHs communicate a need to feel respected and cared for by the management [137–140]. To provide compassionate, attentive, and sensitive health care, a working culture characterized by a caring and respecting management [141, 142]. Hence, to facilitate self-transcendence and meaning, a sound and health-promoting working culture should be facilitated and nurtured [143, 144]. Both NH residents and NH staff will benefit from transforming the traditional institutional model of care into a responsive, patient-centered, homelike approach [131].

## Conclusion

This study indicates that nurse-patient-interaction directly relates to NH residents’ inter- and intrapersonal self-transcendence and meaning-in-life. It seems obligatory that high-quality nurse-patient-interaction should be developed to foster NH residents’ sense of worthiness, self-acceptance, adjustment, and connectedness, as these might foster self-transcendence, meaning-in-life and thereby wellbeing. The NHs should be developed so that the staff nurses have more time for interacting with their residents; continuity and mutuality in nurse-patient relationships should be prioritized and facilitated.

## Data Availability

The datasets generated and/or analysed during the current study are not publicly available due to Norwegian Act on medical and health research (ACT 2008–06-20 no. 44):§ 38 but are available from the corresponding author on reasonable request. All raw data is in Norwegian.

## Abbreviations

χ^2^: Chi-square
α: Cronbach’s alpha reliability coefficient
ρc: Composite Reliability coefficient
CFI: Comparative Fit Index
JoLNH: A nursing home which is certified as a Joy-of-Life Nursing Home
NH: Nursing home
QoL: Quality of life
MSc: Academic degree Master
NPI: Nurse-Patient Interaction
NPIS: Nurse-Patient Interaction Scale
PIL: The questionnaire termed “Purpose-In-Life test” assessing perceived meaning-in-life
RN: Registered Nurse
RMSEA: Root Mean Square Error of Approximation
SEM: Structural Equation Modelling
SD: Standard Deviation
SRMS: Standardized Root Mean Square Residual
ST: Self-Transcendence
STS: Self-Transcendence Scale
ST1: Inter-personal Self-Transcendence
ST2: Intra-personal Self-Transcendence
TLI: Tucker Lewis Index
